# SPAG5‐AS1 inhibited autophagy and aggravated apoptosis of podocytes via SPAG5/AKT/mTOR pathway

**DOI:** 10.1111/cpr.12738

**Published:** 2020-01-19

**Authors:** Jun Xu, Yujie Deng, Yi Wang, Xiaofang Sun, Shuqin Chen, Guoxiang Fu

**Affiliations:** ^1^ Department of Geriatrics Shanghai Jiao Tong University Affiliated Sixth People's Hospital Shanghai China; ^2^ Department of Endocrinology The Affiliated Hospital of Qingdao University Qingdao China; ^3^ Department of Nephrology Yueyang Hospital of Integrated Traditional Chinese and Western Medicine Shanghai University of Traditional Chinese Medicine Shanghai China; ^4^ Department of Endocrinology and Metabolism Ningbo First Hospital Ningbo China

**Keywords:** AKT/mTOR, autophagy, diabetic nephropathy, podocyte injury, SPAG5, SPAG5‐AS1

## Abstract

**Objectives:**

Podocyte injury is a prediction marker of diabetic nephropathy (DN), and AKT/mTOR pathway–mediated inhibition of autophagy is widely reported to contribute to podocyte damage. Recent study stated that sperm‐associated antigen 5 (SPAG5) activated AKT/mTOR signalling in bladder urothelial carcinoma, indicating SPAG5 might regulate autophagy and play a role in podocyte damage.

**Materials and methods:**

Apoptosis and autophagy of human podocytes (HPCs) were detected by flow cytometry and immunofluorescence (IF). Gene level was assessed by Western blot and RT‐qPCR. Molecular interactions were determined by pulldown, RNA immunoprecipitation (RIP), co‐immunoprecipitation (co‐IP), chromatin immunoprecipitation (ChIP) and luciferase reporter assays.

**Results:**

SPAG5 mRNA and protein levels were upregulated under high glucose treatment in HPCs. Silencing SPAG5 reversed the increase of apoptosis and decrease of autophagy in high glucose–treated HPCs. Later, we found a long non‐coding RNA (lncRNA) SPAG5 antisense RNA1 (SPAG5‐AS1) as a neighbour gene to SPAG5. Mechanistically, YY1 transcriptionally upregulated SPAG5‐AS1 and SPAG5 in high glucose–treated podocytes. SPAG5‐AS1 acted as a competitive endogenous RNA (ceRNA) to regulate miR‐769‐5p/YY1 axis and induce SPAG5. SPAG5‐AS1 interacted with ubiquitin‐specific peptidase 14 (USP14) and leads to de‐ubiquitination and stabilization of SPAG5 protein.

**Conclusions:**

This study revealed that SPAG5‐AS1 inhibited autophagy and aggravated apoptosis of podocytes via SPAG5/AKT/mTOR pathway, indicating SPAG5‐AS1/SPAG5 as a potential target for the alleviation of podocyte injury and offering new thoughts for the treatments of DN.

## 1 INTRODUCTION

Diabetic nephropathy is a prevalent complication of diabetes and results in end‐stage kidney disease.^1^ Clinical characteristics of DN include persistent albuminuria elevation, glomerular basement membrane (GBM) thickening, as well as extracellular matrix aggregation, leading to the block of autophagy flux, injury of podocyte and progressive dysfunction of renal.^2^

Podocyte injury is considered as a clinical predictor in the development of DN.^3^, ^4^ Autophagy of podocytes helps the maintenance of cell homeostasis through protein and organelle degradation, and the dysfunction of autophagy is referred to as a contributing factor of podocyte injury.^5^, ^6^ For example, Zhou et al pointed out that autophagy inhibitor aggravated the injury of podocytes, indicating podocyte autophagy as a target for lupus renal protection.^7^ Jin et al revealed that exosomes derived from adipose‐derived stem cells alleviated diabetic nephropathy by promoting autophagy flux and attenuating apoptosis in podocyte.^8^ However, the mechanism underlying the dysfunction of podocyte autophagy remains to be better understood.

It is well established that AKT/mTOR pathway hinders autophagy in a diversity of cell types, such as cancer cells,^9^ cardiomyocytes^10^ and also podocytes.^8^ It is demonstrated that high glucose leads to the activation of phosphoinositide 3‐kinase (PI3K)/Akt signalling pathway.^11^ Also, studies have proved that activating mTOR signalling accelerates podocyte damage in DN patients, suggesting that mTOR signalling–mediated dysfunction of autophagy is tightly linked to DN disease.^12^, ^13^ SPAG5 is known to be a mitotic spindle–associated protein^14^ and regulated the growth of multiple types of cancer cells.^15^, ^16^ Recently, a study showed that sperm‐associated antigen 5 (SPAG5) activated AKT/mTOR pathway in bladder urothelial carcinoma,^17^ indicating that SPAG5 might regulate autophagy through AKT/mTOR signalling and potentially play a role in podocyte injury. However, never has SPAG5 been related to autophagy or podocyte damage yet.

Long non‐coding RNAs (lncRNAs) are a group of non‐protein coding RNAs longer than 200 nucleotides deeply involved in a wide spectrum of human diseases.^18^, ^19^, ^20^ A number of works have established the correlation of lncRNAs with DN. For example, lncRNA‐Gm4419 silence alleviated NF‐κB/NLRP3 inflammasome‐regulated inflammation in DN.^21^ Knockdown of lncRNA plasmacytoma variant translocation 1 (PVT1) inhibited podocyte damage and apoptosis in through upregulating forkhead box A1 (FOXA1).^22^ LINC01619 regulated miR‐27a/FOXO1 to trigger oxidative stress and podocyte injury in DN.^23^ SPAG5‐AS1 was discovered by our study as a neighbour gene to SPAG5 through browsing UCSC, indicating that SPAG5 might be regulated by SPAG5‐AS1. However, SPAG5‐AS1 has never been related to SPAG5 and podocyte injury.

Therefore, this study planned to detect whether SPAG5 played a role in high glucose (HG)–induced podocyte damage through AKT/mTOR pathway and how SPAG5 expression was regulated in podocyte injury.

## 2 MATERIALS AND METHODS

### 2.1 Cell culture and treatment

The conditionally immortalized human podocyte cell line was purchased from the University of Bristol Medical School (Bristol, UK) and incubated on flasks based on the supplier's protocol. RPMI‐1640 Medium (Sigma‐Aldrich) with 10% foetal bovine serum (Gibco BRL) and penicillin‐streptomycin solution (Sigma‐Aldrich) were utilized for culturing podocyte at 37°C in 5% CO_2_. Besides, human podocyte was treated with 5.5 mmol/L of D‐glucose (termed NG group), 5.5 mmol/L of D‐glucose plus 24.5 mmol/L of mannitol (termed MA group) or 30 mmol/L of D‐glucose (termed HG group) for 48 hours. MHY1485 (10 μmol/L), the mTOR activator, was bought from Sigma‐Aldrich for treating cells.

### 2.2 RNA extraction and real‐time quantitative PCR (RT‐qPCR)

Total cellular RNA was isolated as per the user guide of TRIzol (Invitrogen) and reverse‐transcribed into complementary DNA (cDNA) by the use of PrimeScript RT Reagent Kit (Takara). RT‐qPCR was conducted with the SYBR Premix Ex Taq™ (Takara) for the gene fold change. The relative quantitative result was analysed by the comparative 2^−ΔΔCt^ method and normalized to GAPDH or U6.

### 2.3 Western blotting

The cellular protein extracts were acquired in RIPA lysis buffer (Santa Cruz Biotechnology) and then subjected to electrophoresis on 10% SDS‐PAGE gel. Following the transfer of membrane onto PVDF, protein samples were probed with the primary antibodies including anti‐SPAG5 (ab200671; Abcam), anti‐c‐Caspase 3 (ab2302; Abcam), anti‐t‐Caspase 3 (ab13847; Abcam), anti‐c‐Caspase 9 (ab2324; Abcam), anti‐t‐Caspase 9 (ab32539; Abcam), anti‐Bax (ab32503; Abcam), anti‐Bcl‐2 (ab32124; Abcam), anti‐LC‐3I/II (ABC929, Millipore, St. Charles, MI, USA), anti‐p62 (ab56416), anti‐Beclin1 (ab210498), anti‐p‐AKT (thr30; ab105731), anti‐p‐AKT (ser473; ab81283), anti‐t‐AKT (ab179463), anti‐p‐mTOR (ser2448; ab109268), anti‐t‐mTOR (ab2732), anti‐YY1 (ab109228), anti‐USP14 (ab192618), anti‐p‐USP14 (S432; sc‐393872, Santa Cruz Biotechnology) and anti‐GAPDH. After washing in PBS and incubation with secondary antibodies, membrane was visualized with enhanced chemiluminescence (ECL) detection system (Santa Cruz Biotechnology).

### 2.4 Immunofluorescence (IF) staining

The fixed human podocyte in 4% paraformaldehyde was mixed with 0.1% Triton X‐100 for 15 minutes, blocked in 5% bovine serum albumin (BSA) for 1 hour. Cells were treated with the following primary antibodies against anti‐podocin (P0372; Sigma‐Aldrich Co), SPAG5, USP14 and LC‐3 (ProteinTech Group) at 4°C overnight, with secondary antibodies at 37℃ for 1 hour. After DAPI staining, laser confocal microscopy was used for observation.

### 2.5 Plasmid transfection

The gene silencing transfection plasmids including sh‐SPAG5#1/2, sh‐SPAG5‐AS1#1/2, sh‐YY1 and miR‐769‐5p inhibitor, as well as their relative negative controls (sh‐NC and NC inhibitor), were all purchased from Ribo Biotechnology Co. Ltd. The overexpression plasmids including pcDNA3.1/USP14, pcDNA3.1/SPAG5 and miR‐769‐5p mimic, as well as the empty pcDNA3.1 vector and NC mimic as controls, were all produced at GeneCopoeia. Cells were transfected with these plasmids for 48 hours in accordance with the instructions of Lipofectamine 3000 reagent (Invitrogen).

### 2.6 Cell apoptosis analysis

Human podocyte was initially digested in 0.25% trypsin without EDTA, centrifuged for 5 minutes and re‐suspended in PBS. Cells were then incubated with 300 μL of binding buffer and dyed with 5 μL of Annexin V‐FITC following with 10 μL of PI. Apoptotic cells were detected by flow cytometry (BD Biosciences).

### 2.7 Fluorescence in situ hybridization (FISH)

The SPAG5‐AS1 FISH probe was designed and produced by Ribo Biotechnology. The subcellular localization of SPAG5‐AS1 was assayed by FISH kit as suppliers' request. Cells in 24‐well plates with glass slides were fixed in 4% paraformaldehyde and permeabilized with 0.5% Triton X‐100, followed by adding hybridization buffer and probe. Finally, cells were treated with DAPI and observed.

### 2.8 GFP‐mRFP‐LC3 adenoviral transfection

Human podocytes were plated on the glass‐bottomed culture dishes and then infected with the adenoviral vectors which contained GFP‐mRFP‐LC3 (HanBio Technology). Thereafter, culture mediums were substituted with fresh medium and HPCs underwent incubation for 1 day. The HPCs were then observed by using a confocal laser scanning microscope (LSM 880 with Airyscan; Zeiss) to monitor the autophagy flux, and numbers of yellow and green dots were evaluated.

### 2.9 Nucleus‐cytoplasm separation

Nucleus‐cytoplasm separation was carried out using the Subcellular Protein Fraction Kit (Thermo Fisher Scientific). Cell lysates were reaped and cultured on ice. After centrifugation, nuclear RNA was isolated from the supernatant containing the cytoplasmic RNA which was then removed to a clean tube. The expression levels of GAPDH, U6 and SPAG5‐AS1 in nuclear and cytoplasmic fractions of human podocytes were measured by RT‐qPCR.

### 2.10 Dual‐luciferase reporter analysis

For gene promoter luciferase analysis, the human podocytes were plated into 24‐well plates and co‐transfected with the pGL3‐Basis luciferase vector (Promega) containing SPAG5‐AS1/SPAG5 promoter, the indicated silencing transfection plasmids (sh‐SPAG5‐AS1 or sh‐YY1), along with the pRL‐TK‐Renilla vector (Promega) as normalization. The possible miR‐769‐5p binding sites to SPAG5‐AS1 sequence or 3′‐UTR of YY1 were predicted by bioinformatics. SPAG5‐AS1 or YY1 fragments containing wild‐type (Wt) or mutant (Mut) miR‐769‐5p binding sites were inserted into pmirGLO reporter vector (Promega) and named as SPAG5‐AS1 WT/Mut or YY1 WT/Mut, followed by the co‐transfection of miR‐769‐5p mimic or NC mimic into cells, respectively. Reporter gene assay was conducted after 48 hours using Dual Luciferase Assay System (Promega).

### 2.11 Stability assay

Confluent HPCs in 6‐well plates were cultivated with 20 μg/mL of actynomicin D (ActD) or 40 μg/mL of cycloheximide (CHX) (both from Sigma‐Aldrich) to block mRNA transcription and protein synthesis. Cells were finally collected for total RNA and protein isolation to perform mRNA stability and protein stability analysis.

### 2.12 Pull‐down assay

After in vitro transcription with the application of Ribo™ RNAmax‐T7 biotin‐labelled transcription Kit (Ribo Biotechnology), biotin‐labelled RNAs were incubated with cell lysates and magnetic beads. The RNA enrichment was evaluated by RT‐qPCR, Western blotting or silver staining.

### 2.13 Chromatin immunoprecipitation (ChIP)

ChIP assay was performed according to the protocol of EZ‐ChIPTM Chromatin Immunoprecipitation Kit (Millipore). The chromatin fragments of 200‐500 bp were immunoprecipitated with specific antibodies or negative control anti‐IgG antibody. The DNA released from the protein‐DNA complex was collected by beads and purified for RT‐qPCR analysis.

### 2.14 RNA immunoprecipitation (RIP)

RIP assay was processed using EZMagna RIP Kit (Millipore) and anti‐YY1 antibody and anti‐Ago2 antibody following the guidelines provided by supplier. Antibodies against SNRNP70 and IgG were taken as the positive control and negative control, respectively. The silver staining and mass spectrometry analysis were followed.

### 2.15 Co‐immunoprecipitation (Co‐IP)

The lysates of human podocytes were extracted via IP lysis buffer, cultivated with the indicated antibodies and negative control IgG all night at 4°C, followed by incubation with secondary antibody IPKine HRP AffiniPure Mouse Anti‐Rabbit IgG Light Chain (Abbkine, A25022). After mixing with beads, the complex was rinsed in IP buffer for 3 times and boiled with SDS buffer, followed by elution and Western blotting.

### 2.16 Statistical analyses

The experimental results were all acquired from three independent replications and analysed using Prism 6 statistical package (GraphPad). Data were presented as means ± SD. Statistical difference was determined by Student's *t* tests and one‐way ANOVA, with *P‐*value < .05 as threshold.

## 3 RESULTS

### 3.1 SPAG5 silence attenuated apoptosis and induced autophagy in high glucose–treated podocytes

**Figure 1:**
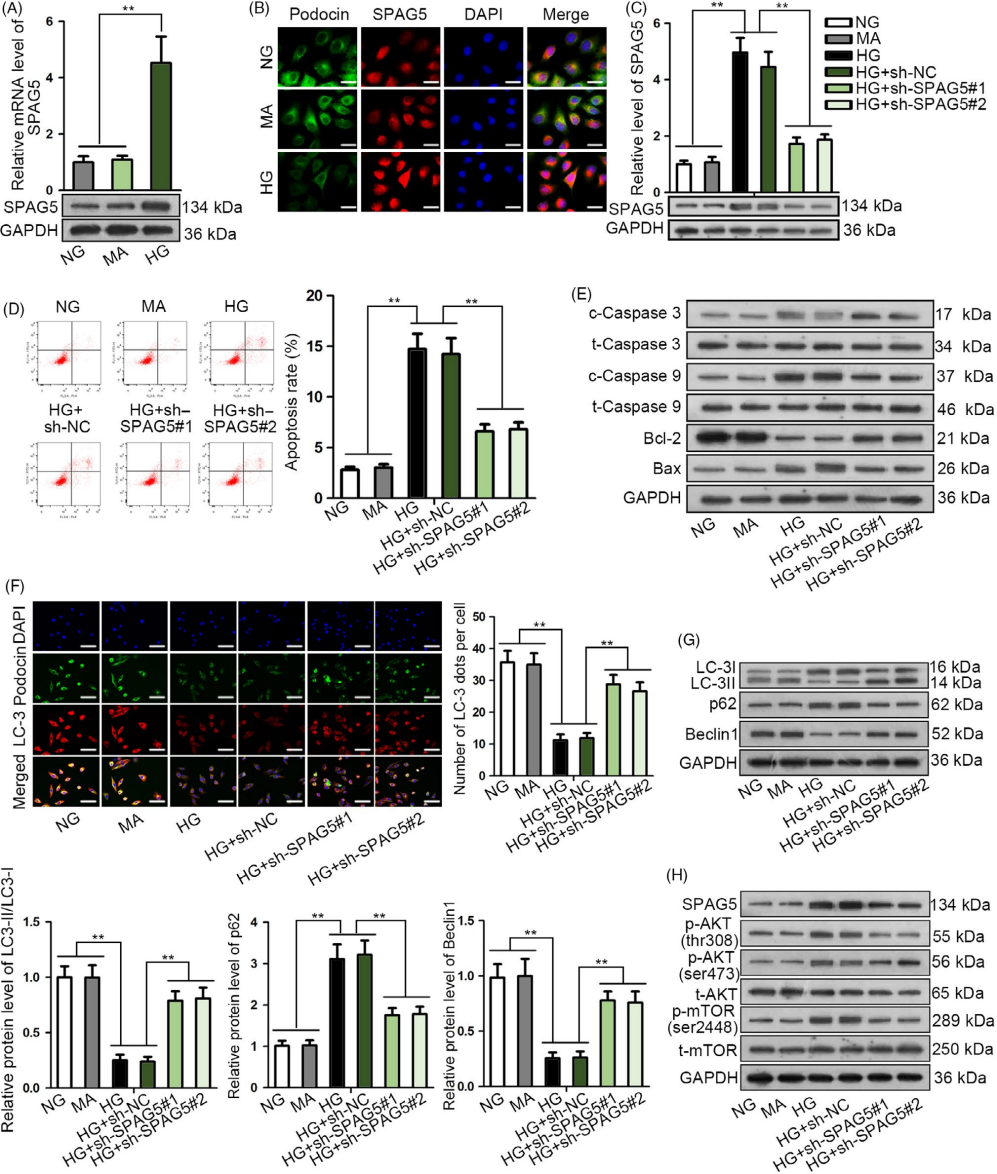
SPAG5 silence attenuated apoptosis and induced autophagy in high glucose–treated podocytes. A, RT‐qPCR and Western blot data for the SPAG5 level in HPCs under HG treatment compared with the control groups (NG or MA treatment). B, SPAG5 and podocin (podocyte marker) fluorescence intensity under HG treatment versus NG and MA controls was tested by IF staining. Scale bar: 25 μm. C, HPCs were treated with HG, MA or HG, and the HG‐treated HPCs were transfected with sh‐NC or sh‐SPAG5#1/2. RT‐qPCR and Western blots of SPAG5 mRNA and protein in HPCs with indicated treatments and transfections. D, Flow cytometry analysis of apoptotic HPCs with indicated treatments and transfections. E, Apoptosis‐related genes were detected by Western blot in HPCs with indicated treatments and transfections. F‐G, IF staining of LC‐3 and podocin, and Western blotting of the autophagy‐related proteins in HPCs with indicated treatments and transfections. Scale bar: 50 μm. H, The protein levels of SPAG5 and phosphorylated and total AKT and mTOR in HPCs with indicated treatments and transfections. All experiments were conducted in triplicates. Data are presented as mean ± SD. **P < .01

First, we detected whether SPAG5 participated in high glucose–induced podocyte damage. RT‐qPCR and Western blot data depicted that compared with the control groups (NG or MA treatment), mRNA and protein levels of SPAG5 were increased under high glucose treatment (HG) (Figure 1A). Accordingly, IF staining showed that SPAG5 presented higher fluorescence intensity in HPCs under HG treatment versus control (Figure 1B). Also, the fluorescence intensity of podocin, the podocyte marker, decreased under HG treatment in HPCs compared to controls (Figure 1B). These data indicated that SPAG5 was potentially involved in HG‐induced podocyte injury. To test the influence of SPAG5, we confirmed the knockdown of SPAG5 at mRNA and protein levels by two sh‐RNAs in HG‐treated HPCs (Figure 1C). Later, we observed that compared to NG and MA treatments, HG induced apoptosis of HPCs, and knocking down of SPAG5 attenuated apoptosis in HG‐treated HPCs (Figure 1D). Apoptosis‐related genes were detected by Western blot. HG treatment in HPCs induced the levels of cleaved caspase 3, cleaved caspase 9 and Bax, whereas reduced the level of Bcl‐2, and silencing SPAG5 in HG‐treated HPCs reversed such phenomenon (Figure 1E). The fluorescence intensities of LC‐3 and podocin in HPCs were reduced by HG treatment, and were restored by the depletion of SPAG5 (Figure 1F). We then detected autophagic flux through infecting HPCs with GFP‐mRFP‐LC3 adenovirus. Consequently, HG treatment decreased the red and yellow puncta compared to control, and such effect was contracted by silencing SPAG5 in HPCs (Figure S1A), suggesting that SPAG5 knockdown restored autophagic flux that was inhibited by HG in HPCs. Besides, we confirmed that SPAG5 knockdown reversed the decrease of LC‐3II/LC‐3I and Beclin1 levels and the increase of p62 levels in HG‐treated HPCs (Figure 1G). Moreover, we validated that SPAG5 depletion counteracted the inductive effect of HG on the levels of SPAG5, p‐AKT (thr308), p‐AKT (ser473) and p‐mTOR (ser2448) in HPCs, with the total levels of AKT and mTOR unchanged (Figure 1H), indicating that SPAG5 positively regulated AKT/mTOR signalling in HG‐treated HPCs. Altogether, these results suggested that SPAG5 silence attenuated apoptosis, induced autophagy and suppressed AKT/mTOR pathway in high glucose–treated HPCs.

### 3.2 SPAG5‐AS1 positively regulated SPAG5 transcription and protein stability in HPCs

**Figure 2:**
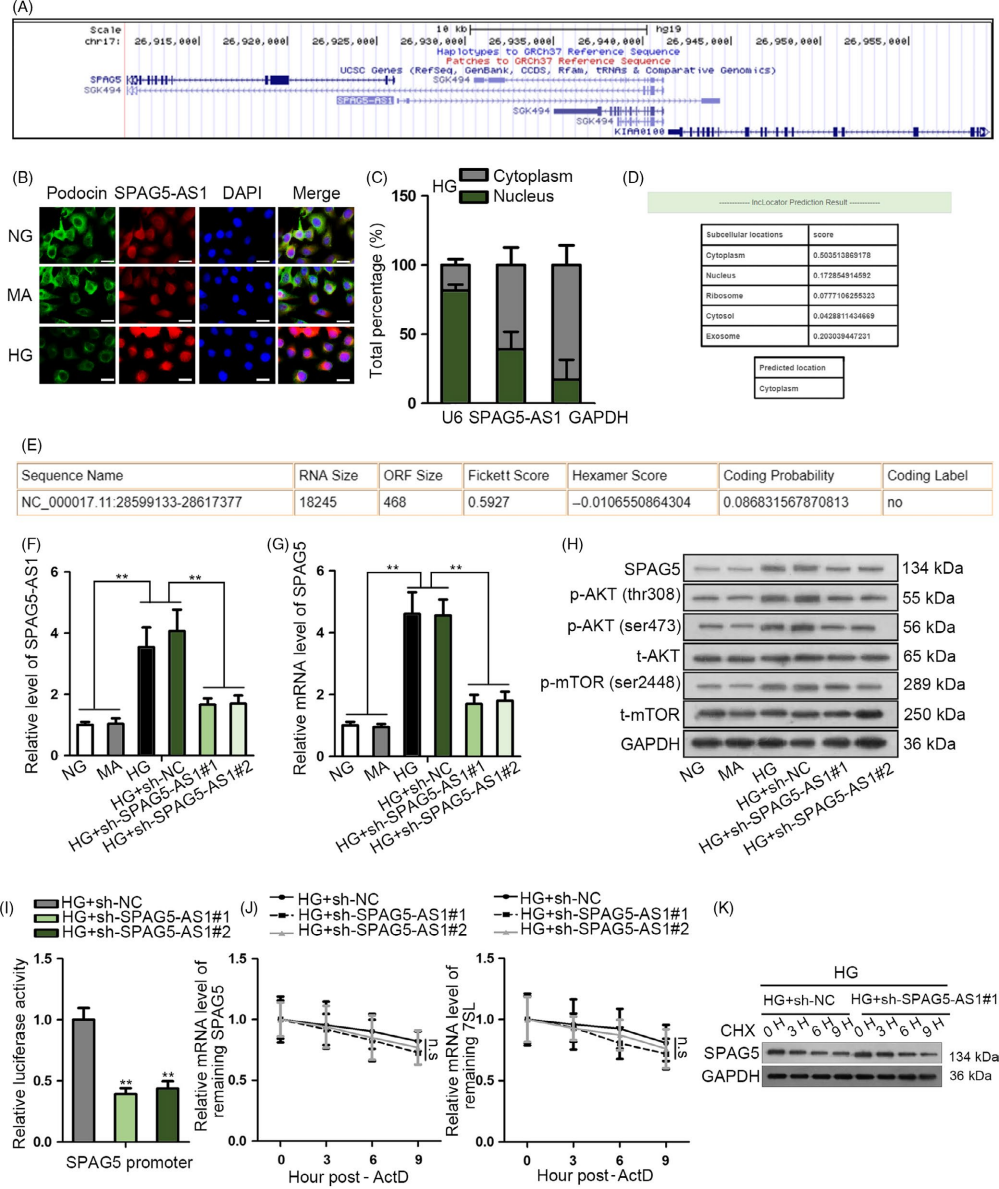
SPAG5‐AS1 positively regulated SPAG5 transcription and protein stability in HPCs. A, UCSC showed that genomic location of SPAG5‐AS1 was neighbour to SPAG5. B‐C, Abundance and localization of SPAG5‐AS1 were determined by FISH and nucleus‐cytoplasm separation assay in HPCs under HG treatment compared to the control groups. Expression of podocyte marker podocin in HPCs was detected by IF staining. Scale bar: 25 μm. D, Bioinformatics tool lncLocator predicted the SPAG5‐AS1 cytoplasmic localization. E, Result of online coding potential analysis tool CPAT for SPAG5‐AS1. F‐G, The mRNA levels of SPAG5‐AS1 and SPAG5 in HG‐treated HPCs after inhibiting SPAG5‐AS1. H, Western blot analysis for the regulation of SPAG5‐AS1 depletion on levels of SPAG5 and phosphorylated and total AKT and mTOR under HG treatment. I, Luciferase activity of SPAG5 promoter reporter in response to the decrease in SPAG5‐AS1 level was monitored under HG treatment. J‐K, The ActD and CHX treatments were used for detecting SPAG5 mRNA stability and the protein degradation of SPAG5 caused by sh‐SPAG5‐AS1 transfection in HG‐treated HPCs. All experiments were conducted in triplicates. Data are presented as mean ± SD. **P < .01

Next, we tried to explore how SPAG5 expression was regulated in HG‐induced podocyte damage. Through UCSC genome browser (http://genome.ucsc.edu/), we discovered that lncRNA SPAG5‐AS1 was neighbour to SPAG5 (Figure 2A). Their neighbouring genome location indicated that SPAG5‐AS1 might be related to SPAG5, because multiple studies have showed that lncRNAs presented a potential strong correlation with their neighbour genes.^24^, ^25^ FISH and IF staining revealed that SPAG5‐AS1 increased whereas podocin decreased in HPCs under HG treatment compared to the control groups and that SPAG5‐AS1 was mainly located in the cytoplasm of HPCs (Figure 2B). Subcellular fractionation analysis also confirmed that SPAG5‐AS1 level was higher in cytoplasm than in the nucleus of HG‐treated HPCs (Figure 2C). In accordance, results of the online bioinformatics tool lncLocator (http://www.csbio.sjtu.edu.cn/bioinf/lncLocator/) also showed the predominant localization of SPAG5‐AS1 in cytoplasm (score: 0.504) (Figure 2D). Additionally, the online coding potential analysis tool CPAT (http://lilab.research.bcm.edu/cpat/) showed that the open reading frame (ORF) size of SPAG5‐AS1 was 468, and the coding probability was 0.087, indicating that SPAG5‐AS1 was a non‐coding RNA (ncRNA) (Figure 2E). Then, we tried to detect whether SPAG5‐AS1 regulated SPAG5. We validated through RT‐qPCR that SPAG5‐AS1 level was upregulated by HG treatment in HPCs and was significantly silenced by the transfection of sh‐SPAG5‐AS1#1/2 (Figure 2F). The mRNA level of SPAG5 induced by HG in HPCs was impaired by the knockdown of SPAG5‐AS1 (Figure 2G). Western blot analysis depicted that levels of SPAG5 and phosphorylated AKT (thr308 and ser473) and mTOR (ser2448) were induced by HG, and impaired by SPAG5‐AS1 knockdown in HPCs (Figure 2H), indicating that SPAG5‐AS1 positively regulated SPAG5/AKT/mTOR axis in HG‐treated HPCs. Since lncRNAs could regulate their target genes at different levels such as transcriptional level, post‐transcriptional level and post‐translational level,^26^, ^27^, ^28^ we detected at which level SPAG5‐AS1 regulated SPAG5 expression. Luciferase activity of SPAG5 promoter reporter was attenuated by the knockdown of SPAG5‐AS1 silence in HG‐treated HPCs (Figure 2I). However, the half‐life of SPAG5 mRNA stability presented no significant variation under SPAG5‐AS1 depletion compared to control in HG‐treated HPCs (Figure 2J). The protein degradation of SPAG5 was monitored after the addition of CHX, the inhibitor of protein production. Results showed that silence of SPAG5‐AS1 facilitated the degradation of SPAG5 protein in HG‐treated HPCs (Figure 2K). Collectively, these results indicated that SPAG5‐AS1 positively regulated SPAG5 expression by inducing its transcription and protein stability.

### 3.3 SPAG5‐AS1 knockdown attenuated HG‐induced podocyte damage by inhibiting apoptosis and inducing autophagy

**Figure 3:**
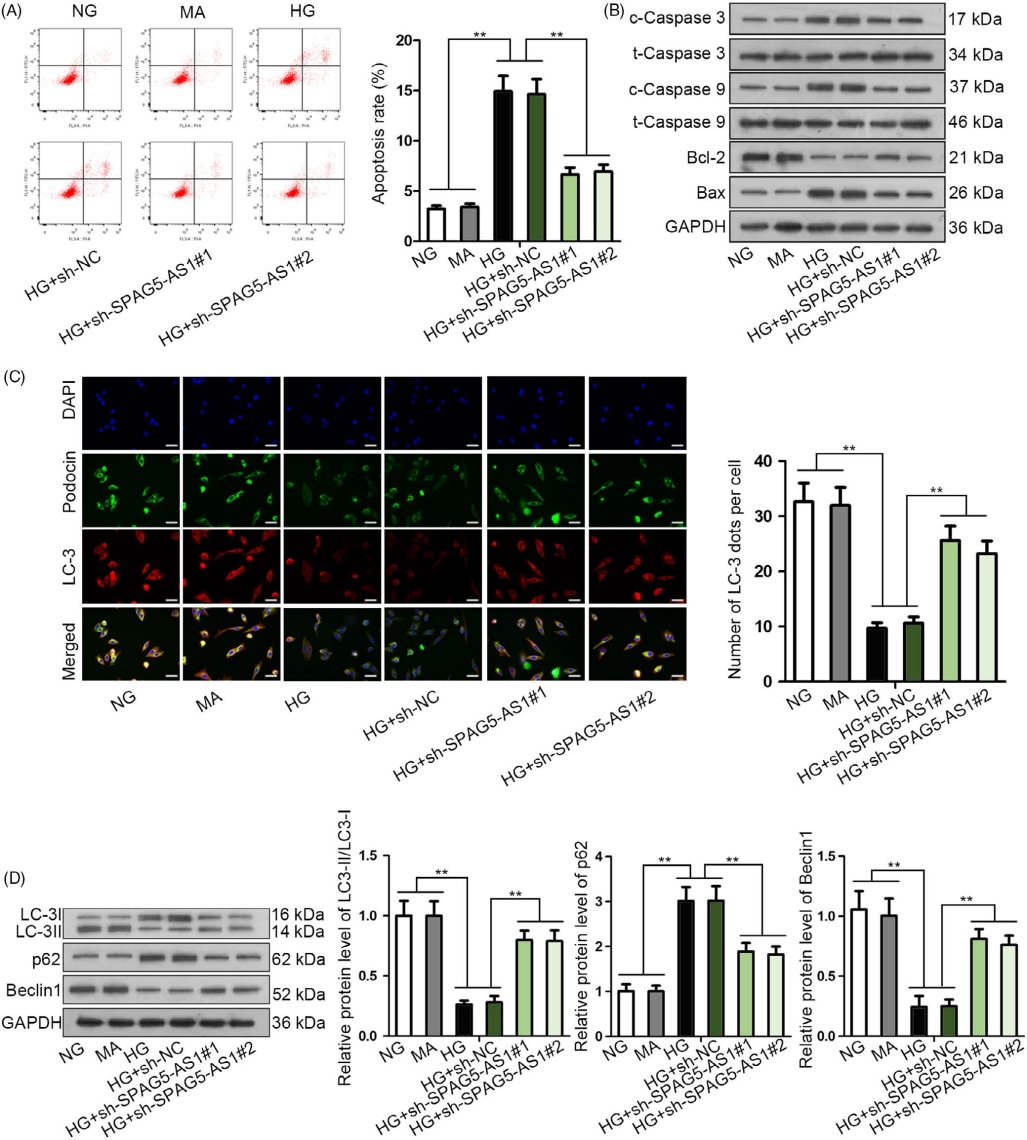
SPAG5‐AS1 knockdown attenuated HG‐induced podocyte damage by inhibiting apoptosis and inducing autophagy. A‐B, HPCs were treated with NG, MA, HG, HG + sh‐NC or HG + sh‐SPAG5‐AS1#1/2. Flow cytometry and Western blot of apoptosis‐related genes were conducted to evaluate HPCs apoptosis for each group. C‐D, Results of IF staining for LC3 and podocin and Western blotting for autophagy‐associated proteins in each group. Scale bar: 50 μm. All experiments were conducted in triplicates. Data are presented as mean ± SD. **P < .01

The function of SPAG5‐AS1 in HG‐induced damage of HPCs was examined as well. As shown by the results of flow cytometry analysis, knockdown of SPAG5‐AS1 countervailed the pro‐apoptotic effect of HG treatment in HPCs (Figure 3A). Silence of SPAG5‐AS1 impaired the high expression of cleaved caspase 3, cleaved caspase 9 and Bax and reversed the low expression of Bcl‐2 in HG‐treated HPCs (Figure 3B). Depletion of SPAG5‐AS1 recovered the fluorescence intensity of LC‐3 and podocin that were reduced by HG treatment in HPCs (Figure 3C). The GFP‐mRFP‐LC3 assay exhibited that silencing SPAG5‐AS1 restored the autophagic flux that was blocked by HG in HPCs (Figure S1B). The decrease of LC‐3II/LC‐3I and Beclin 1 levels and increase of p62 level under HG treatment were reversed by SPAG5‐AS1 knockdown in HPCs (Figure 3D). Jointly, these results implied that SPAG5‐AS1 knockdown attenuated the HG‐induced podocyte damage by inhibiting apoptosis and inducing autophagy.

### 3.4 YY1 transcriptionally upregulated SPAG5‐AS1/SPAG5 in HG‐treated HPCs

**Figure 4:**
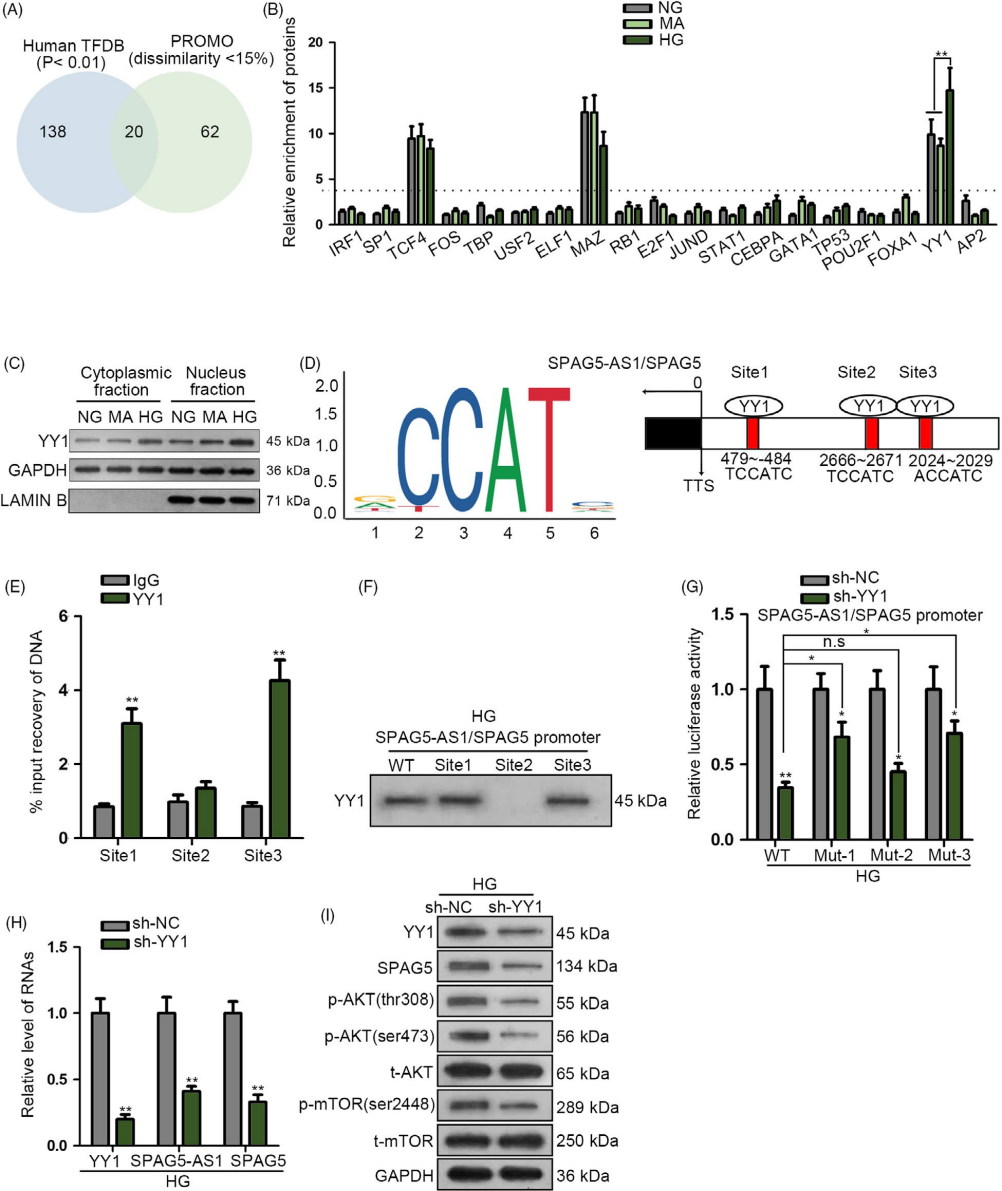
YY1 transcriptionally upregulated SPAG5‐AS1/SPAG5 in HG‐treated HPCs. A‐B, 20 transcription factors (TFs) potentially binding to the promoter of SPAG5‐AS1/SPAG5 according to the prediction of human TFDB and PROMO were presented by Venn diagram and analysed via pull‐down analysis under NG, MA or HG treatment. C, Western blot analysis of the YY1 level under NG, MA or HG treatment in cytoplasmic or nuclear fraction of HPCs. D, 3 YY1 binding sites on the promoter of SPAG5‐AS1/SPAG5 were predicted by JASPAR tool. E, ChIP analysis for the DNA fragments with binding site 1, 2 or 3 in the precipitated products of YY1. F, YY1 protein was detected by Western blot in the pulldown of WT SPAG5‐AS1/SPAG5 promoter and SPAG5‐AS1/SPAG5 promoter with site 1, 2 or 3 in HG‐treated HPCs. G, Luciferase reporter analysis elucidated the influence of the YY1 silence on the luciferase activity of SPAG5‐AS1/SPAG5 promoter reporter of each group. H, RT‐qPCR result for the levels of YY1 mRNA, SPAG5‐AS1 and SPAG5 mRNA responding to knockdown of YY1 in HG‐treated HPCs. I, Western blot of YY1, SPAG5, p‐AKT (tthr308 and ser2448), t‐AKT, p‐mTOR and t‐mTOR of each group. All experiments were conducted in triplicates. Data are presented as mean ± SD. *P < .05, **P < .01

According to the data in UCSC genome browser, SPAG5‐AS1 and SPAG5 shared the same promoter (Figure 2A). Therefore, we deduced that certain transcription factor (TF) was responsible for the co‐transactivation of SPAG5 and SPAG5‐AS1. We obtained the promoter sequence of SPAG5‐AS1/SPAG5 from UCSC and analysed the potential TFs for it in Human TFDB (http://bioinfo.life.hust.edu.cn/HumanTFDB/#!/) (threshold: *P* < .01) and PROMO (http://alggen.lsi.upc.es/cgi-bin/promo_v3/promo/promoinit.cgi?dirDB=TF_8.3) (threshold: dissimilarity ≤ 15%). Combining the prediction results of two bioinformatics tools, we identified 20 TFs potentially binding to the promoter of SPAG5‐AS1/SPAG5 (Figure 4A). Pull‐down analysis revealed that transcription factor 4 (TCF4), MYC‐associated zinc finer protein (MAZ) and Yin Yang 1 (YY1) were enriched in the pulldown of SPAG5‐AS1/SPAG5 promoter; however, only the enrichment of YY1 was increased under HG treatment compared to control in HPCs (Figure 4B).

Yin Yang 1 is a well‐known transcription activator, and former study has demonstrated that high glucose facilitated the translocation of YY1 from cytoplasm to the nucleus of tubular epithelial cells and aggravated diabetes.^29^ Hence, we deduced that HG treatment might also influence YY1 in HPCs and therefore the binding between YY1 and SPAG5‐AS1/SPAG5 promoter was increased, and this observation indicated that YY1 might be responsible for the HG‐caused induction of SPAG5‐AS1 and SPAG5 in HPCs. Expectedly, Western blot analysis confirmed that under HG treatment, the level of nuclear YY1 was increased. Interestingly, the level of YY1 in the cytoplasm of HPCs was also increased under HG (Figure 4C), suggesting that YY1 expression was induced under HG in HPCs. Through JASPAR tool (http://jaspar.genereg.net/), we identified 3 YY1 binding sites on the promoter of SPAG5‐AS1/SPAG5 (Figure 4D). ChIP analysis illustrated that the DNA fragments with binding sites 1 and 3, rather than site 2, were enriched in the precipitated products of YY1 (Figure 4E). In concordance, in HG‐treated HPCs, YY1 protein was detectable in the pulldown of WT SPAG5‐AS1/SPAG5 promoter and SPAG5‐AS1/SPAG5 promoter with site 1 or site 3; however, SPAG5‐AS1/SPAG5 promoter with only site 2 failed to pull down YY1 (Figure 4F). Luciferase reporter analysis elucidated that in HG‐treated HPCs, the silence of YY1 reduced the luciferase activity of SPAG5‐AS1/SPAG5 promoter, and mutation of site 1 or site 3, instead of site 2, could partly restore the luciferase activity (Figure 4G). These data proved that YY1 targeted the promoter of SPAG5‐AS1/SPAG5 at site 1 and site 3.

Later, we confirmed that knockdown of YY1 reduced the levels of YY1 mRNA, SPAG5‐AS1 and SPAG5 mRNA (Figure 4H). Also, protein levels of YY1, SPAG5, p‐AKT (thr308 and ser473) and p‐mTOR (ser2448) were reduced by YY1 knockdown in HG‐treated HPCs, with total AKT and mTOR levels unchanged (Figure 4I). In collection, YY1 transcriptionally upregulated SPAG5‐AS1/SPAG5 in HG‐treated HPCs.

### 3.5 SPAG5‐AS1 induced SPAG5 level through acting as a ceRNA regulating miR‐769‐5p/YY1

**Figure 5:**
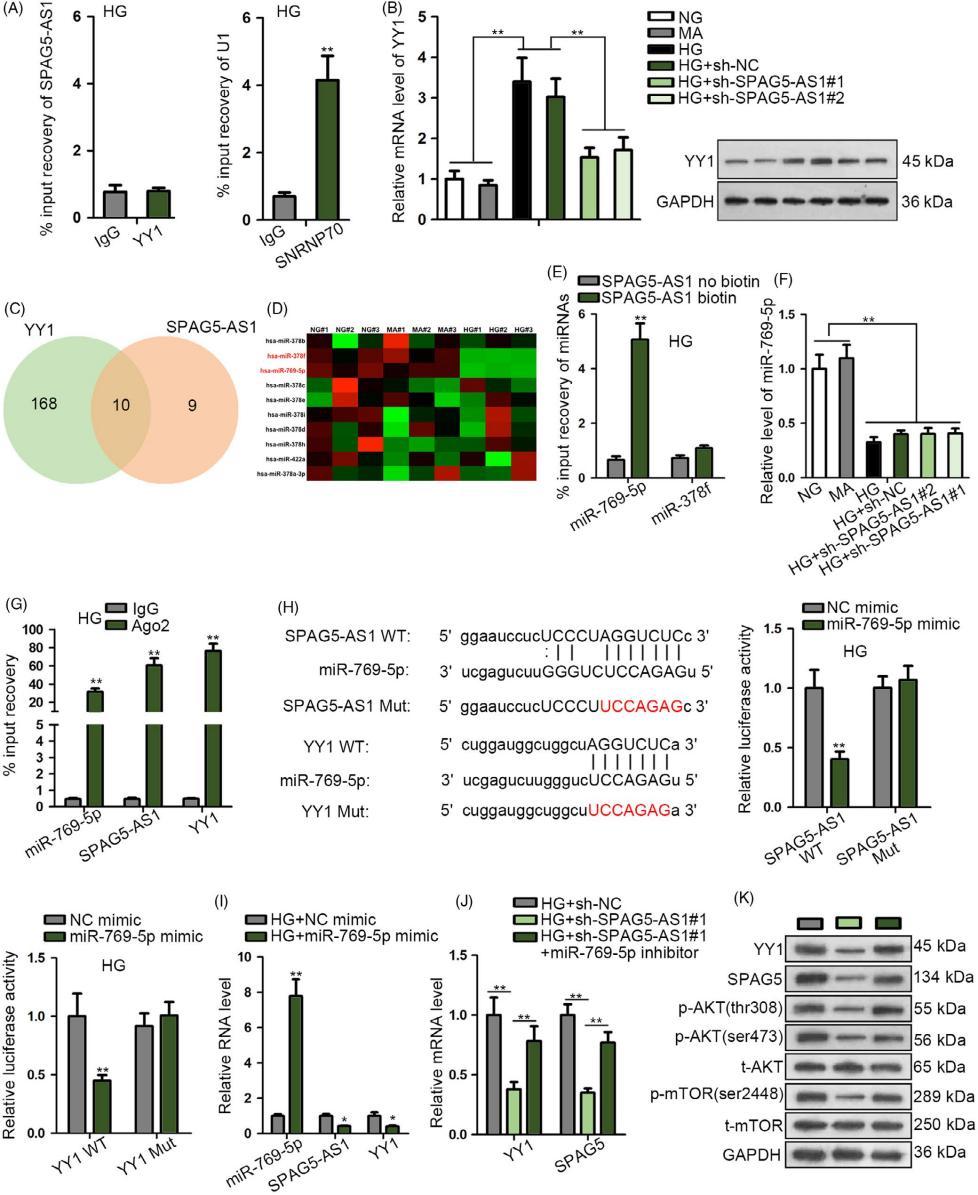
SPAG5‐AS1 induced SPAG5 level through acting as a ceRNA regulating miR‐769‐5p/YY1. A, RIP analysis showed the non‐existence of SPAG5‐AS1 in the YY1 precipitates in HG‐treated HPCs. SNRNP70 and U1 were positive controls. B, YY1 mRNA and protein levels in HPCs treated with NG, MA, HG, HG + sh‐NC or HG + sh‐SPAG5‐AS1#1/2. C‐D, 10 miRNAs potently shared by YY1 and SPAG5‐AS1 were showed as Venn diagram and subjected to RT‐qPCR analysis under the treatment of HG compared to HG and MA in HPCs. E, RNA pull‐down assay was applied for the miR‐769‐5p enrichment in complex pulled down by SPAG5‐AS1 biotin probe or no‐biotin probe. F, MiR‐769‐5p level in each group after repressing SPAG5‐AS1 expression. G, RIP analysis was used to confirm the levels of miR‐769‐5p, SPAG5‐AS1 and YY1 in Ago2 precipitates. H, The wild‐type (WT) and mutant (Mut) binding sites of miR‐769‐5p on SPAG5‐AS1 and YY1 were utilized to conduct luciferase reporter assay to assess the luciferase activity of SPAG5‐AS1 WT and YY1 WT in HG‐treated HPCs after transfection with miR‐769‐5p mimic or NC mimic. I, Levels of miR‐769‐5p, SPAG5‐AS1 and YY1 in HPCs treated with HG + NC mimic or HG + miR‐769‐5p mimic. J, HG‐treated HPCs were transfected with sh‐NC, sh‐SPAG5‐AS1#1 or sh‐SPAG5‐AS1#1 + miR‐796‐5p inhibitor. RT‐qPCR data of the level of YY1 and SPAG5 mRNA of each group. K, Western blot of the protein levels of YY1, SPAG5, p‐AKT (thr308 and ser473) and p‐mTOR (ser2448) in HG‐treated HPCs with indicated transfection. All experiments were conducted in triplicates. Data are presented as mean ± SD. *P < .05, **P < .01

Subsequently, we explored the mechanism behind the regulation of SPAG5 expression by SPAG5‐AS1. Since we have observed that SPAG5‐AS1 positively regulated SPAG5 transcription in HG‐treated HPCs, and that YY1 transcriptionally activated SPAG5, we deduced that SPAG5‐AS1 regulated SPAG5 transcription through YY1. RIP analysis showed that SPAG5‐AS1 was not enriched in the YY1 precipitates (Figure 5A), indicating that there was no interaction between SPAG5‐AS1 and YY1 protein. Also, we validated that YY1 mRNA and protein levels were induced by HG in HPCs and silencing SPAG5‐AS1 impaired YY1 mRNA and protein levels (Figure 5B). Previously, we validated that SPAG5‐AS1 was predominantly located in the cytoplasm of HPCs. Cytoplasmic lncRNAs can function as ceRNAs to competitively interact with miRNAs.^30^, ^31^ Therefore, we hypothesized that SPAG5‐AS1 upregulated YY1 expression through sponging certain miRNA.

Via Starbase3.0 (http://starbase.sysu.edu.cn/), we identified 10 miRNAs potently shared by YY1 and SPAG5‐AS1 (Figure 5C). RT‐qPCR data depicted that among the 10 miRNAs, only miR‐769‐5p and miR‐378f exhibited significant downregulation under the treatment of HG compared to HG and MA in HPCs (Figure 5D), indicating the participation of the 2 miRNAs in HG‐induced HPC injury. However, pull‐down assay showed that only miR‐769‐5p was pulled down by SPAG5‐AS1 biotin probe rather than no‐biotin probe (Figure 5E), indicating that miR‐769‐5p was a target for SPAG5‐AS1. Moreover, we confirmed that the silence of SPAG5‐AS1 had no influence on the expression of miR‐769‐5p in HG‐treated HPCs (Figure 5F). RIP analysis confirmed that miR‐769‐5p was co‐immunoprecipitated with SPAG5‐AS1 and YY1 by Ago2 antibody (Figure 5G). The predicted miR‐769‐5p sites on SPAG5‐AS1 and YY1 were substituted with complementary sequences to generate SPAG5‐AS1 Mut and YY1 Mut reporters (Figure 5H). Luciferase reporter assay confirmed that miR‐769‐5p overexpression abrogated luciferase activity of SPAG5‐AS1 WT and YY1 WT in HG‐treated HPCs (Figure 5H).

RT‐qPCR analysis confirmed that miR‐769‐5p mimic induced miR‐769‐5p expression and reduced YY1 and SPAG5‐AS1 expressions in HG‐treated HPCs (Figure 5I, Figure S2A). It was probable that miR‐769‐5p mimic reduced SPAG5‐AS1 level because miR‐769‐5p reduced YY1 expression so that weakened the YY1‐mediated transactivation of SPAG5‐AS1. Thereafter, we validated that in HG‐treated HPCs, miR‐769‐5p inhibitor countervailed the repressive effect of sh‐SPAG5‐AS1#1 on YY1 and SPAG5 mRNA levels (Figure 5J, Figure S2B). Western blot confirmed that inhibiting miR‐769‐5p impaired the repressive effect of SPAG5‐AS1 silence on the levels of YY1, SPAG5, p‐AKT (thr308 and ser473) and p‐mTOR (ser2448) in HG‐treated HPCs, with total AKT and mTOR unchanged (Figure 5K, Figure S2C). In a word, data above indicated that SPAG5‐AS1 induced SPAG5 level through acting as a ceRNA regulating miR‐769‐5p/YY1.

### 3.6 SPAG5‐AS1 stabilized SPAG5 protein through interacting with USP14 in HG‐treated HPCs

**Figure 6:**
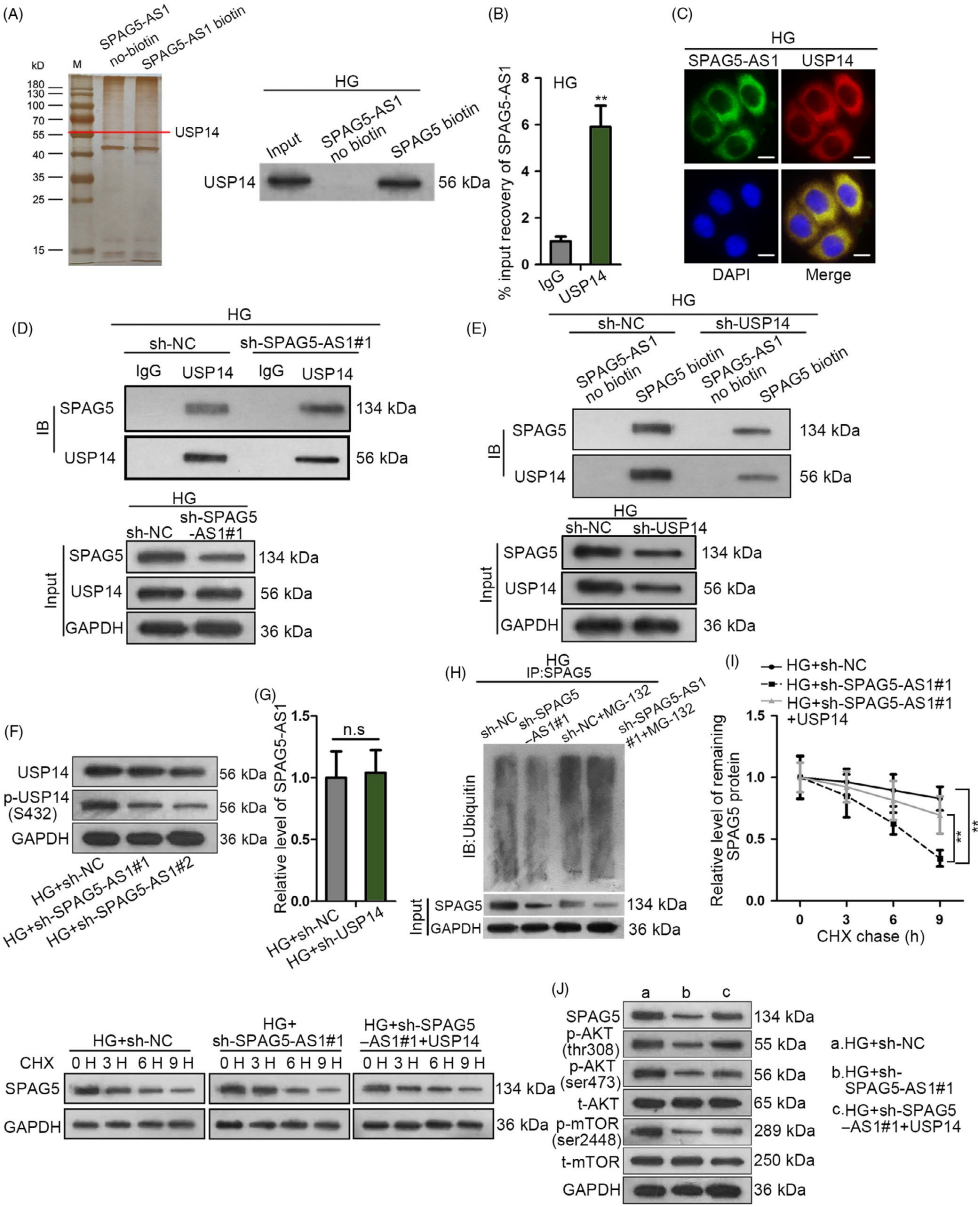
SPAG5‐AS1 stabilized SPAG5 protein through interacting with USP14 in HG‐treated HPCs. A, The silver staining gel manifested that a protein band in the pull‐down products of SPAG5‐AS1 biotin group was observed in HG‐treated HPCs. Western blot assay for the USP14 enrichment in the pulldown of SPAG5‐AS1 biotin group and SPAG5‐AS1 no‐biotin group in HG‐treated HPCs. B, RIP analysis confirmed the SPAG5‐AS1 level in USP14‐binding complexes. C, FISH and IF staining were performed for the localization of SPAG5‐AS1 and USP14 protein in HG‐treated HPCs. Scale bar: 10 μm. D, Co‐IP assay of the SPAG5 and USP14 enrichments in the precipitated products of anti‐USP14, under SPAG5‐AS1 depletion or sh‐NC control. E, Western blot for the SPAG5 and USP14 abundance in the pulldown of SPAG5‐AS1 biotin group and no‐biotin group under USP14 knockdown or sh‐NC control. F, Silence of SPAG5‐AS1 reduced the level of p‐USP14 (S432) without changing its total protein level in HG‐treated HPCs. G, Knockdown of USP14 failed to impact SPAG5‐AS1 level in HG‐treated HPCs. H, The immunoblot of ubiquitin in the precipitates of SPAG5 and the input level of SPAG5 under the silence of SPAG5‐AS1 in HG‐treated HPCs treated with or without MG‐132. I, Remaining SPAG5 protein level after the treatment of CHX was detected by Western blot and quantitated at 0, 3, 6 and 9 h in HG‐treated HPCs transfected with sh‐NC, sh‐SPAG5‐AS1#1 or sh‐SPAG5‐AS1#1 + pcDNA3.1/USP14. J, The protein level of SPAG5 as well as phosphorylated and total levels of AKT and mTOR in HPCs treated with HG + sh‐NC, HG + sh‐SPAG5‐AS1#1 or HG + sh‐SPAG5‐AS1#1 + USP14. All experiments were conducted in triplicates. Data are presented as mean ± SD. **P < .01

Furthermore, we interrogated how SPAG5‐AS1 regulated SPAG5 protein stability. As shown in the silver staining gel, we obtained a protein band in the pull‐down products of SPAG5‐AS1 biotin group rather than SPAG5‐AS1 no‐biotin group in HG‐treated HPCs (Figure 6A). By analysing the band by mass spectrometry, we found that USP14 was a SPAG5‐AS1‐interacting protein. Western blot assay confirmed the enrichment of USP14 in the pulldown of SPAG5‐AS1 biotin group instead of SPAG5‐AS1 no‐biotin group in HG‐treated HPCs (Figure 6A). USP14 is a regulator of de‐ubiquitination and has been reported to be activated through AKT/mTOR pathway and negatively regulate autophagy by K48 de‐ubiquitination in neurodegenerative diseases.^32^ Hence, we speculated that SPAG5‐AS1 might regulate SPAG5 through USP14.

RNA immunoprecipitation analysis confirmed that SPAG5‐AS1 was abundant in USP14‐binding complexes (Figure 6B). FISH and IF staining confirmed that SPAG5‐AS1 and USP14 protein were co‐localized in cytoplasm in HG‐treated HPCs (Figure 6C). Co‐IP assay demonstrated that in HG‐treated HPCs, SPAG5 was enriched in the precipitated products of anti‐USP14, and silencing SPAG5‐AS1 reduced such enrichment, with the input level of SPAG5 reduced and input level of USP14 unchanged (Figure 6D), indicating that SPAG5‐AS1 mediated the binding of USP14 to SPAG5. Pull‐down assay illustrated that SPAG5 and USP14 were both enriched in the pulldown of SPAG5‐AS1 biotin group rather than no‐biotin group, and the silence of USP14 reduced such enrichment, with input levels of USP14 and SPAG5 decreased (Figure 6E), indicating that USP14 was required for the interaction between SPAG5‐AS1 and SPAG5.

In addition, former study stated that activating AKT induced phosphorylation and activation of USP14.^32^ Since we have proved that SPAG5‐AS1 positively regulated AKT/mTOR pathway in HG‐treated HPCs, we tried to test whether SPAG5‐AS1 could induce USP14 phosphorylation. As expected, the silence of SPAG5‐AS1 reduced the level of p‐USP14 (S432) without changing its total protein level in HG‐treated HPCs (Figure 6F). We also confirmed that knockdown of USP14 failed to impact SPAG5‐AS1 level in HG‐treated HPCs (Figure 6G). Then, we validated that the silence of SPAG5‐AS1 increased the ubiquitination of SPAG5 protein in HG‐treated HPCs (Figure 6H). Overexpression of USP14 counteracted the facilitative effect of SPAG5‐AS1 knockdown on SPAG5 protein degradation in HG‐treated HPCs (Figure 6I, Figure S2D). These data indicated that SPAG5‐AS1 regulated the de‐ubiquitination of SPAG5 through USP14. Overexpression of USP14 restored the levels of SPAG5, p‐AKT (thr308 and ser473) and p‐mTOR (ser2448) that were reduced by SPAG5‐AS1 silence in HG‐treated HPCs, with total AKT and mTOR unchanged (Figure 6J, Figure S2E). Together, it was suggested that SPAG5‐AS1 stabilized SPAG5 protein through interacting with USP14 in HG‐treated HPCs, and that USP14 and AKT/mTOR formed a positive feedback loop.

### 3.7 SPAG5‐AS1 promoted apoptosis and attenuated autophagy in HG‐treated HPCs through SPAG5/AKT/mTOR pathway

**Figure 7:**
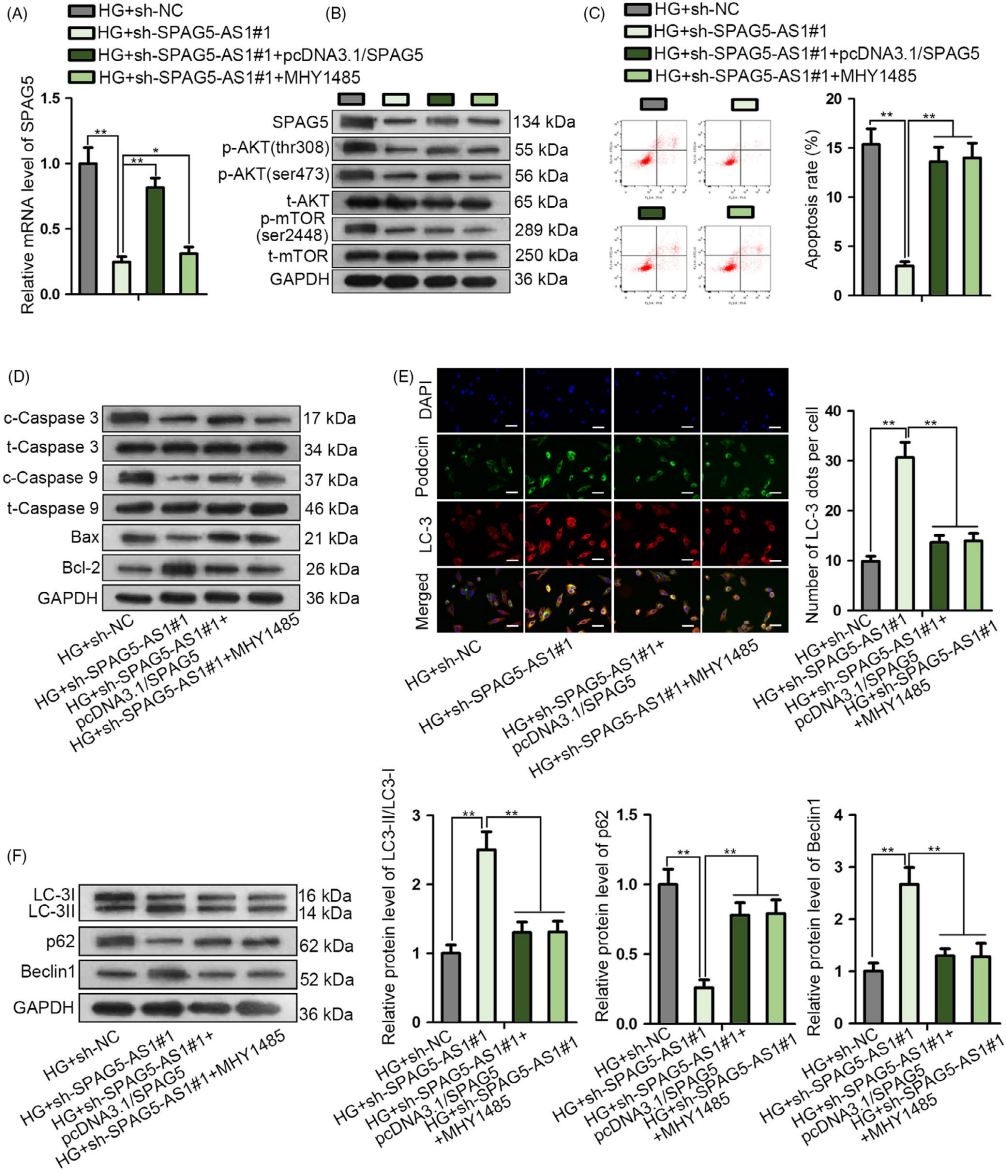
SPAG5‐AS1 promoted apoptosis and attenuated autophagy in HG‐treated HPCs through SPAG5/AKT/mTOR pathway. HPCs treated with HG were transfected with sh‐NC, sh‐SPAG5‐AS1#1, sh‐SPAG5‐AS1#1 + pcDNA3.1/SPAG5 or sh‐SPAG5‐AS1#1 plus the treatment of MHY1485, an activator of mTOR. A‐B, SPAG5 mRNA and protein levels, as well as the phosphorylated and total levels of AKT and mTOR in each group. C‐D, Flow cytometry of apoptotic HPCs and Western blotting results of apoptosis‐related genes in HPCs in each group. E‐F, LC‐3 and podocin fluorescence intensity and autophagy‐related proteins were analysed by IF staining and Western blotting in all groups. Scale bar: 50 μm. All experiments were conducted in triplicates. Data are presented as mean ± SD. *P < .05, **P < .01

Finally, we designed rescue assays to detect whether SPAG5/AKT/mTOR pathway was required for the regulation of SPAG5‐AS1 on HG‐induced HPC damage. HPCs were treated with HG and transfected with sh‐NC, sh‐SPAG5‐AS1#1, sh‐SPAG5‐AS1#1 + pcDNA3.1/SPAG5, or sh‐SPAG5‐AS1#1 plus the treatment of MHY1485, an activator of mTOR. Both the mRNA and protein levels of SPAG5 reduced by SPAG5‐AS1 silence were restored by pcDNA3.1/SPAG5 transfection rather than MHY1845 treatment (Figure 7A, Figure S3A). As reported, USP14 was activated by AKT,^32^ so the non‐effect of mTOR activator on SPAG5 indicated that it was AKT, rather than mTOR that activated USP14 and led to upregulation of SPAG5 level in HG‐treated HPCs. Also, Western blot analysis confirmed that overexpression of SPAG5 restored p‐AKT (thr308 and ser473) and p‐mTOR (ser2448) that were reduced by SPAG5‐AS1 knockdown, whereas MHY1485 only restored p‐mTOR (ser2448) in HG‐treated HPCs (Figure 7B, Figure S3B).

Flow cytometry analysis presented that either overexpressing SPAG5 or adding MHY1485 restored the apoptosis that was reduced by SPAG5‐AS1 silence in HG‐treated HPCs (Figure 7C, Figure S3C). Overexpression of SPAG5 or addition of MHY1485 reversed the decrease of cleaved caspase 3, cleaved caspase 9, and Bax and increase of Bcl‐2 caused by SPAG5‐AS1 silence in HG‐treated HPCs (Figure 7D, Figure S3D). LC‐3 fluorescence intensity and podocin expression were induced by SPAG5‐AS1 silence and impaired by the co‐transfection of pcDNA3.1/SPAG5 or treatment of MHY1485 (Figure 7E, Figure S3E). Autophagic flux was induced by silencing SPAG5‐AS1 in HG‐treated HPCs, and such effect was countervailed by the co‐transfection of pcDNA3.1/SPAG5 or the treatment of MHY1485 (Figure S3F). The increase of LC‐3II/LC‐3I and Beclin1 and decrease of p62 caused by SPAG5‐AS1 silence were reversed by pcDNA3.1/SPAG5 or activation of mTOR (Figure 7F, Figure S3G). In conclusion, it was suggested that SPAG5‐AS1 promoted apoptosis and attenuated autophagy in HG‐treated HPCs through SPAG5/AKT/mTOR pathway.

## 4 DISCUSSION

Podocyte injury is known to be related to DN evolution and is referred to as a clinical predictor.^3^, ^4^ Studies have demonstrated that dysregulated autophagy is a major reason for podocyte injury,^5^, ^6^ suggesting that autophagy induction could be a potential approach to alleviating podocyte injury and treating DN.^8^ Therefore, exploration of the mechanism underlying autophagy dysfunction in podocytes may offer new potential therapeutic target for DN treatment.

The inhibition of autophagy via AKT/mTOR pathway is largely demonstrated in podocyte injury.^8^ Previous studies illustrated that AKT/mTOR signalling was activated by HG and contributed to podocyte damage and autophagy inhibition in DN.^11^, ^12^, ^13^ These findings suggested that AKT/mTOR is an essential signalling to regulate autophagy and podocyte injury, and that targeting this signalling is a promising way to ameliorate DN progression. Interestingly, a recent study showed that SPAG5 can activate AKT/mTOR pathway in bladder urothelial carcinoma.^17^ SPAG5 has been reported to be an oncogene promoting proliferation in multiple cancer cells, such as hepatocellular cancer and triple‐negative breast cancer.^15^, ^16^ However, its effect on activating AKT/mTOR signalling suggested that SPAG5 might inhibit autophagy through AKT/mTOR and therefore lead to the injury of podocyte. Herein, we firstly revealed that SPAG5 was upregulated in HPCs under HG treatment and silence of SPAG5 reversed the HG‐caused apoptosis induction and autophagy inhibition in HPCs, indicating that targeting SPAG5 could alleviate HG‐induced podocyte damage.

Through UCSC, we discovered a novel lncRNA SPAG5‐AS1 that was neighbour to SPAG5‐AS1. Mounting studies have revealed the strong correlation between antisense RNAs with their neighbour genes.^24^, ^25^ Therefore, we suggested that SPAG5 was regulated by SPAG5‐AS1 in HG‐induced podocyte injury. As expected, we validated that silencing SPAG5‐AS1 attenuated the apoptosis induction and the autophagy inhibition in HG‐treated podocytes. Moreover, we confirmed that SPAG5‐AS1 positively regulated SPAG5 transcription and protein stability.

Mechanistically, we identified that SPAG5‐AS1 and SPAG5 shared promoter according to their genomic localization. Interestingly, we found that the binding of YY1 at SPAG5‐AS1/SPAG5 promoter was enhanced under HG treatment in podocytes. Previous study revealed that high glucose treatment in tubular epithelial cells accelerated nuclear translocation of YY1 and promoted diabetes.^29^ However, we discovered that YY1 expression was not only elevated in nucleus, but also elevated in cytoplasm of HPCs, indicating that the total level of YY1 was elevated in HPCs under HG. Later, we confirmed that YY1 bound to SPAG5‐AS1/SPAG5 promoter and induced SPAG5‐AS1 and SPAG5 expressions in HG‐treated HPCs.

Furthermore, we showed that SPAG5‐AS1 upregulated YY1 through sponging miR‐769‐5p so as to induce SPAG5 transcription. Although lncRNAs are reported to regulate transcription of target genes through recruiting TFs,^33^ our data demonstrated that SPAG5‐AS1 was predominantly located in cytoplasm and cannot interact with YY1 protein. Also, considering that we found that HG treatment induced both cytoplasmic and nucleus level of YY1, we suggested that SPAG5‐AS1 regulated YY1 expression. We sorted out miR‐769‐5p from 10 miRNAs shared by SPAG5‐AS1 and YY1 because it was downregulated by HG in HPCs and interacted with SPAG5‐AS1. Former studies showed that miR‐769‐5p participated in prolonged concussion symptoms and acted a predictor of lung cancer patient survival.^34^, ^35^ However, this was the first time for miR‐769‐5p to be related to HG‐induced podocyte injury. Additionally, we discovered that SPAG5‐AS1 could not regulate miR‐769‐5p expression but miR‐769‐5p inhibited SPAG5‐AS1 expression. We suggested that miR‐769‐5p regulated SPAG5‐AS1 through YY1‐mediated transcription.

Moreover, we demonstrated that SPAG5‐AS1 interacted with USP14 to regulate de‐ubiquitination and stabilization of SPAG5 protein. USP14 is a deubiquitinating enzyme which presents reversible association with proteasome, and can inhibit the proteasome activity via trimming K48 ubiquitin chains on the proteasome‐bound substrates.^36^, ^37^ Recent study revealed that USP14 could be phosphorylated and activated by AKT, and could negatively regulate autophagy in neurodegenerative diseases.^32^, ^38^ Herein, we validated that SPAG5‐AS1 regulated the de‐ubiquitination of SPAG5 relying on USP14, and therefore activated AKT/mTOR signalling. Also, we validated that SPAG5‐AS1 could induce p‐USP14 (ser432). Since SPAG5‐AS1 can activate AKT/mTOR pathway, we suggested that SPAG5‐AS1 induced p‐USP14 (ser432) through AKT. These findings indicated the reciprocal activation between USP14 and AKT mediated by SPAG5‐AS1/SPAG5 in HG‐treated HPCs. Finally, rescue assay suggested that SPAG5‐AS1 knockdown attenuated HG‐induced podocyte injury through SPAG5/AKT/mTOR axis.

## 5 CONCLUSIONS

**Figure 8:**
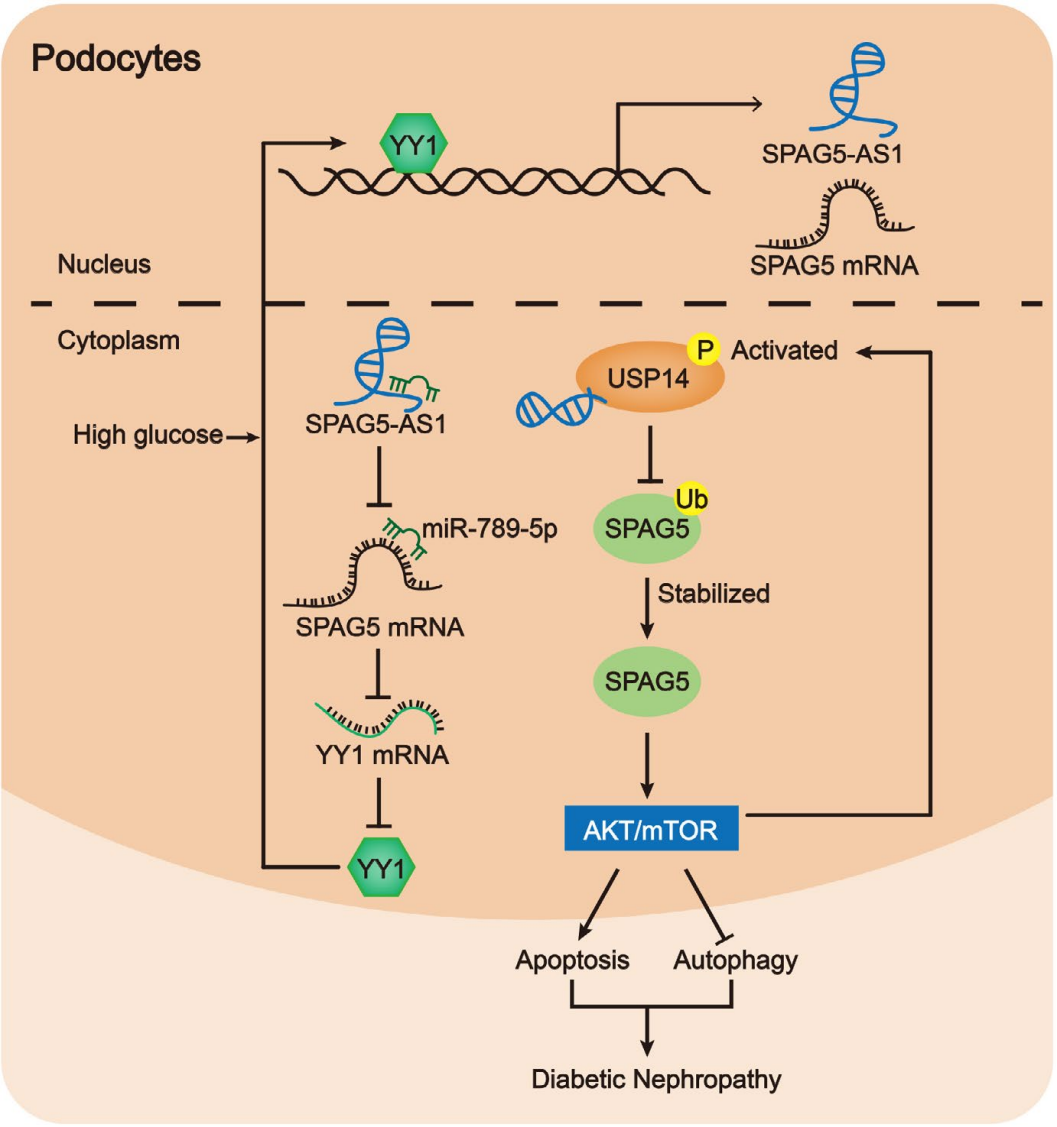
SPAG5‐AS1/miR‐769‐5p/YY1‐positive feedback loop transcriptionally induced SPAG5 and SPAG5‐AS1 interacted with USP14 to induce de‐ubiquitination of SPAG5, so that AKT/mTOR signalling was activated to inhibit autophagy and induce apoptosis in podocytes

This study firstly revealed that SPAG5 positively regulated HG‐induced podocyte injury. Functionally, silencing SPAG5 attenuated apoptosis and induced autophagy in HG‐treated HPCs. SPAG5 was positively regulated by and its neighbour gene SPAG5‐AS1. Mechanistically, YY1 induced transactivation of SPAG5‐AS1/SPAG5. SPAG5‐AS1/miR‐769‐5p/YY1 formed a positive feedback loop to induce SPAG5 transcription and SPAG5‐AS1 stabilized SPAG5 protein through USP14, so that SPAG5‐AS1 activated SPAG5/AKT/mTOR signalling and inhibited autophagy in HPCs (Figure 8). These findings suggested that targeting SPAG5‐AS1/SPAG5 might be a novel approach to alleviating HG‐induced podocyte injury and offered new thoughts to the treatment of DN.

## CONFLICT OF INTEREST

None.

## AUTHOR CONTRIBUTIONS

Jun Xu together with Yujie Deng contributed to manuscript drafting, reviewing as well as revising, and Yi Wang, Xiaofang Sun, Shuqin Chen and Guoxiang Fu were responsible for experiments and data. All authors made great contributions to this study.

## Data Availability

Research data are not shared.
