# Supplementary figures and images for: SPAG5‐AS1 inhibited autophagy and aggravated apoptosis of podocytes via SPAG5/AKT/mTOR pathway
Source: Cell Prolif. 2020 Jan 19;53(2):e12738. doi: 10.1111/cpr.12738 (PMC7046304; doi:10.1111/cpr.12738)

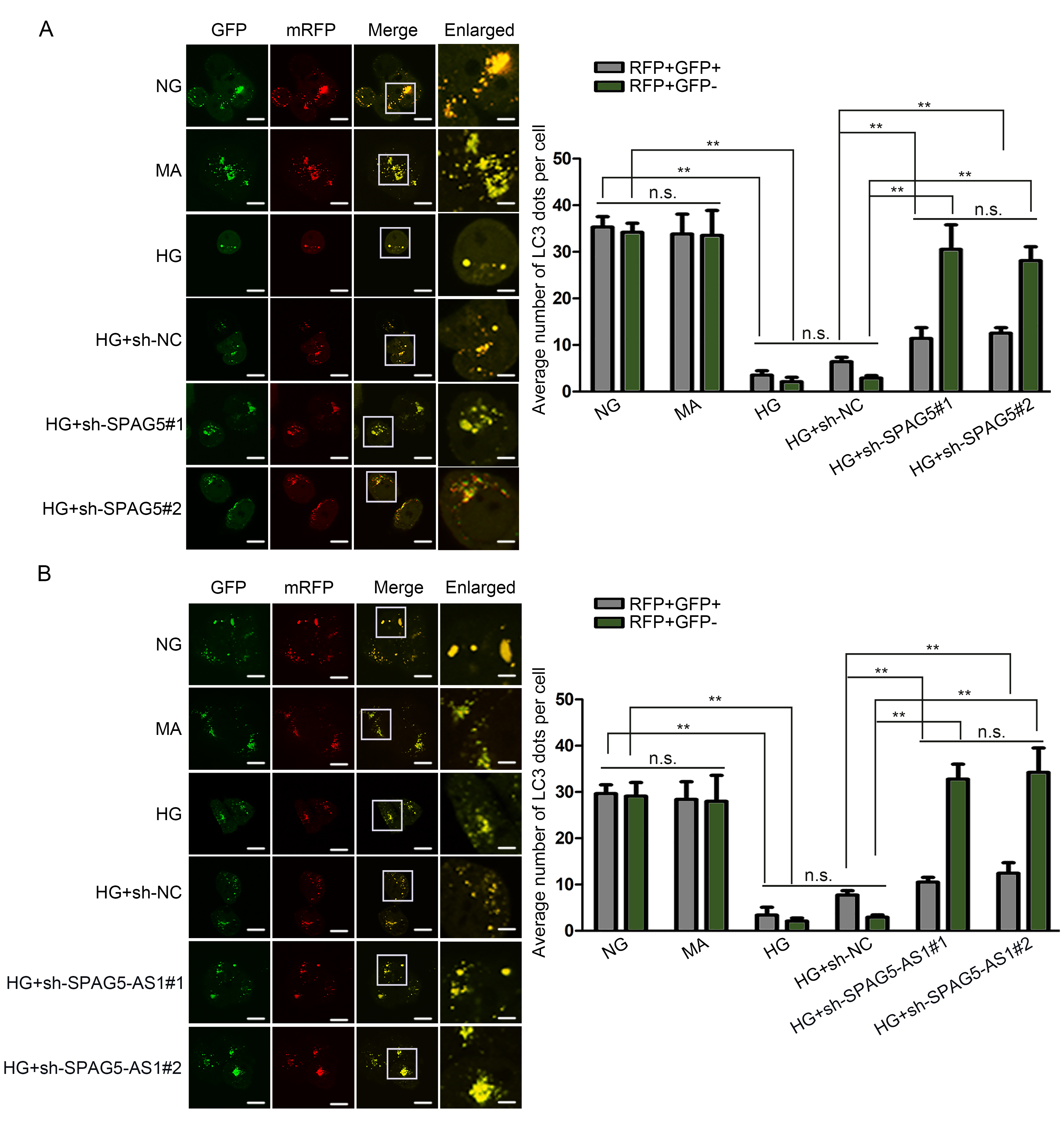

Supplement: Supplementary file 1 [file CPR-53-e12738-s001.jpg]

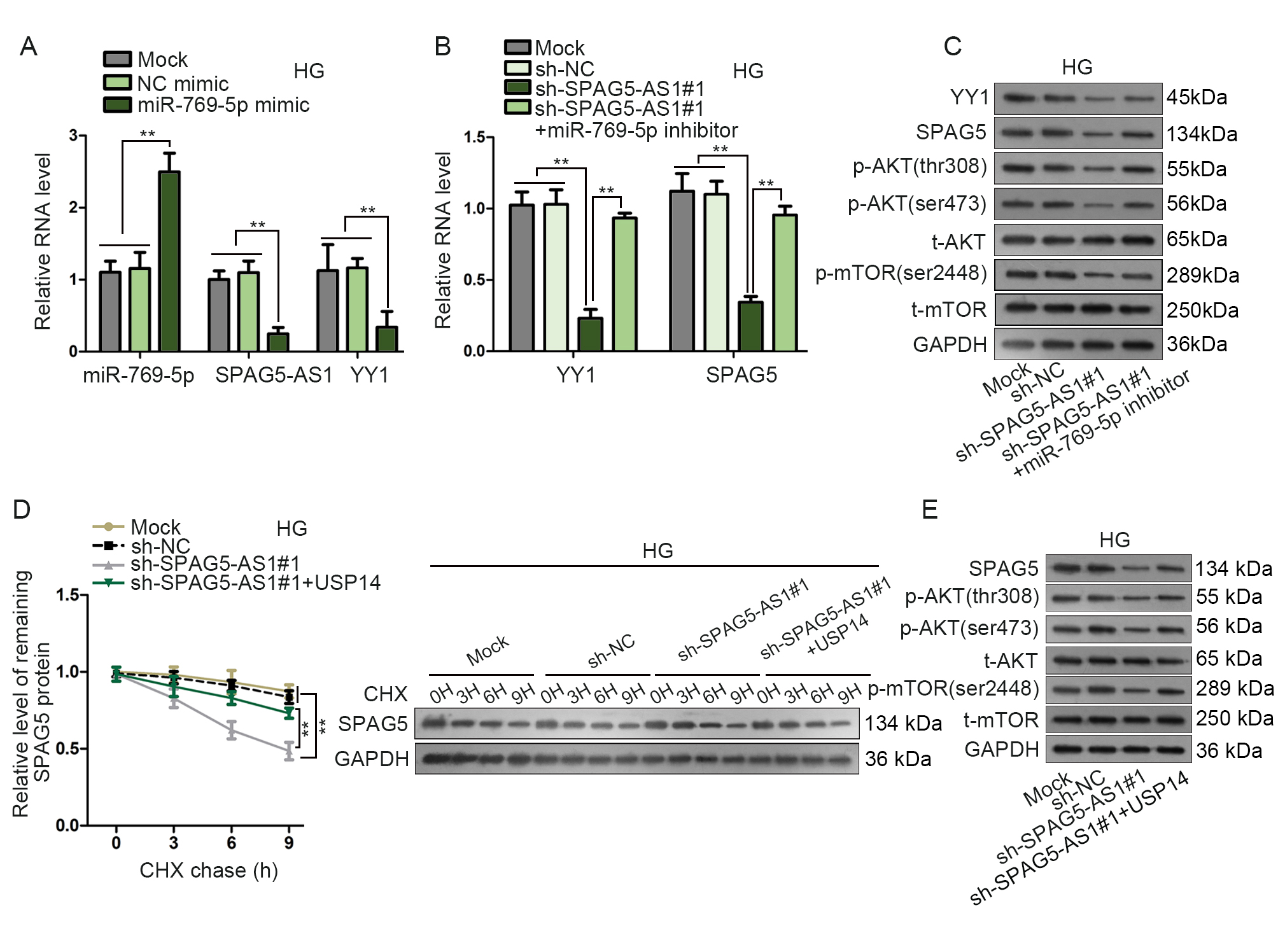

Supplement: Supplementary file 2 [file CPR-53-e12738-s002.jpg]

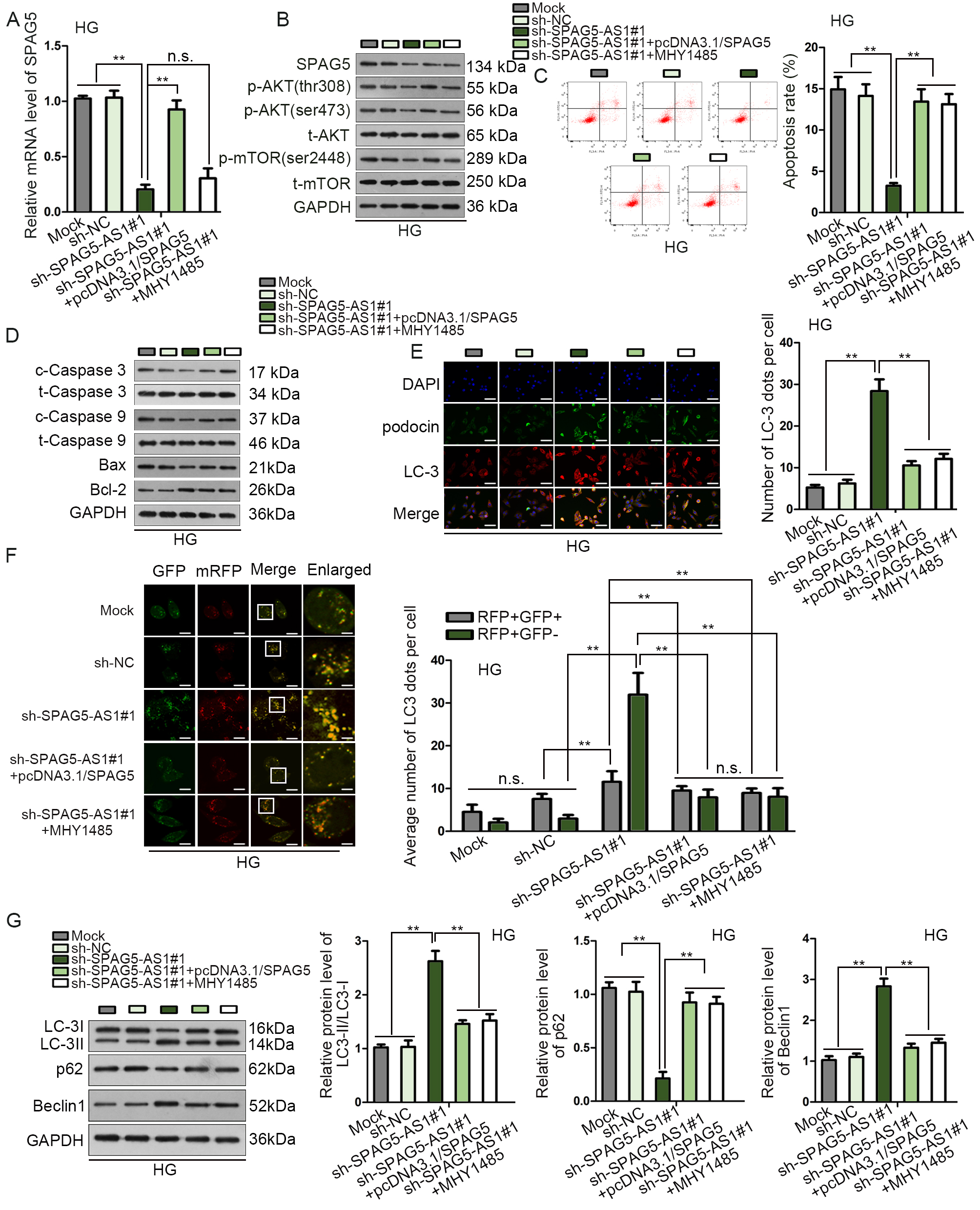

Supplement: Supplementary file 3 [file CPR-53-e12738-s003.jpg]
